# Phthalocyanine Doped Metal Oxide Nanoparticles on Multiwalled Carbon Nanotubes Platform for the detection of Dopamine

**DOI:** 10.1038/srep43181

**Published:** 2017-03-03

**Authors:** Ntsoaki G. Mphuthi, Abolanle S. Adekunle, Omolola E. Fayemi, Lukman O. Olasunkanmi, Eno E. Ebenso

**Affiliations:** 1 Department of Chemistry, School of Mathematical and Physical Sciences, Faculty of Agriculture, Science and Technology, North-West University (Mafikeng Campus), Private Bag X2046, Mmabatho 2735, South Africa; 2 Material Science Innovation & Modelling (MaSIM) Focus Area, Faculty of Agriculture, Science and Technology, North-West University (Mafikeng Campus), Private Bag X2046, Mmabatho 2735, South Africa; 3 Department of Chemistry, Faculty of Science, Obafemi Awolowo University, 220005 Ile-Ife, Nigeria

## Abstract

The electrocatalytic properties of metal oxides (MO = Fe_3_O_4_, ZnO) nanoparticles doped phthalocyanine (Pc) and functionalized MWCNTs, decorated on glassy carbon electrode (GCE) was investigated. Successful synthesis of the metal oxide nanoparticles and the MO/Pc/MWCNT composite were confirmed using UV-Vis, EDX, XRD and TEM techniques. Successful modification of GCE with the MO and their composite was also confirmed using cyclic voltammetry (CV) technique. GCE-MWCNT/ZnO/29H,31H-Pc was the best electrode towards DA detection with very low detection limit (0.75 μM) which compared favourably with literature, good sensitivity (1.45 μA/μM), resistance to electrode fouling, and excellent ability to detect DA without interference from AA signal. Electrocatalytic oxidation of DA on GCE-MWCNT/ZnO/29H,31H-Pc electrode was diffusion controlled but characterized with some adsorption of electro-oxidation reaction intermediates products. The fabricated sensors are easy to prepare, cost effective and can be applied for real sample analysis of dopamine in drug composition. The good electrocatalytic properties of 29H,31H-Pc and 2,3-Nc were related to their (quantum chemically derived) frontier molecular orbital energies and global electronegativities. The better performance of 29H,31H-Pc than 2,3-Nc in aiding electrochemical oxidation of DA might be due to its better electron accepting ability, which is inferred from its lower E_LUMO_ and higher χ.

Dopamine (DA) is a neurotransmitter in mammalian brain tissues that belongs to the family of inhibitory/catecholamine neurotransmitters. It plays an important physiological role in the functioning of central nervous, renal, hormonal and cardiovascular systems as an extra cellular chemical messenger, as well as in drug addiction. Its function is to regulate neural interactions by reducing the permeability of gap junctions between adjacent neurons of the same type^1^. Abnormal DA level may relate to many diseases, such as Parkinson’s disease, Huntington’s disease, Alzheimer’s diseases and tardive dyskinesia^2^, where the dopaminergic activity is lower than in healthy individuals. The opposite is true in schizophrenia where the activity of the dopaminergic neurons is increased due to abnormalities in their regulation. Furthermore, the development of anorexia nervosa and bulimia nervosa has also been associated with altered dopaminergic activities^2^,^3^,^4^. Therefore, it is important to rapidly and accurately measure DA concentrations in biological fluids for clinical diagnosis.

Although DA is an electrochemically active compound on some electrode surfaces, its electrochemical detection in biological fluids using bare electrode is often ineffective. This is mainly because of interfering species such as ascorbic acid (AA) and uric acid (UA)^5^. As a result, the accuracy of its detection is very low in real sample analysis. The basal concentration of DA is 0.01–1 μM, while the concentration of AA and UA is 100–1000 times higher than that of DA, hence it is crucial to develop sensitive and selective methods for determination of DA^4^. A great contribution to disease diagnosis would be a development of electrochemical sensor that would measure DA at low levels of characteristic of living system (26–40 nmol L^−1^)^6^,^7^.

There is currently an interest in the use of metal oxide NPs, metal-doped metal oxides, metal oxide-CNTs (carbon nanotubes) nanocomposites, and metal oxide-polymer composites^8^ in electrochemistry to improve the performance of electrochemical detection of biological and environmental analytes^9^. Analytical devices based on nanostructured metal oxides are cost-effective, highly sensitive due to the large surface-to-volume ratio of the nanostructure and additionally show excellent selectivity^8^. Metal (M) and metal oxides (MO) NPs have been used to modify electrodes for use as electrocatalysts and biosensors; hence they play an important role in diagnostic devices^10^. Shan *et al*.^11^ investigated Fe_3_O_4_/chitosan modified electrode as a feasible sensor for detection of H_2_O_2_. He found that there was limited interference, prompt response, good reproducibility and the electrode showed long term stability. In addition, Adekunle *et al*.^12^ conducted a study on the voltammetric detection of DA using easily prepared nano-scaled iron oxide (Fe_2_O_3_) catalyst supported on MWCNTs modified pyrolytic graphite electrode. Furthermore, Fang^5^ also studied the voltammetric sensing of DA using Fe_3_O_4_ NPs modified gold electrode. The modified electrode gave promoting effect and high stability toward the electrochemical oxidation of DA. Chen *et al*.^13^ suggested that combining good conductivity of ZnO NPs and remarkable properties of CNTs; it could result in potential applications of semiconducting CNTs as novel photocatalysts. Aravind *et al*.^14^ mentioned that because of the great surface area, high electrocatalytic activity, excellent electron transfer rate of CNTs, strong adsorption ability and fast electron transfer kinetics of ZnO, a biosensor was fabricated using ZnO NP-decorated MWCNTs and tested for the selective detection of DA. ZnO NPs have a wide range of use and applications in industrial purposes, solar cells UV light-emitting devices, gas sensors, photocatalysts, pharmaceutical and cosmetic^15^.

Phthalocyanines (Pcs) and their derivatives are well-known blue-green organic semiconductor materials that belong to the family of aromatic heterocyclic conjugated molecules, with alternating single-double bond structures^16^. They are made of delocalised π-electrons system^17^, where π-electron delocalisation and interactions with central mental atom on metal phthalocyanines (MPcs) determine the redox properties^18^. Their thermal and chemical stability are important properties that make them molecules to be incorporated into electrochemical sensors. Furthermore the possibility of incorporating about 70 different metal atoms into their ring^19^, and also being able to have variation of the substituents in the side chain, can allow production of peculiar and optimized thin films having different degrees of sensitivity, selectivity and stability^20^. Wang^21^ reported that among the metal substituted Pc, lead-Pc is the most interesting one in terms of reproducibility, fast response recovery and excellent gas sensitivity. Pcs are capable of binding non-specifically with various analytes through van der Waal forces, hydrogen bonding, and coordination interactions with the central metal^22^. However non-fictionalized Pcs have a low surface-to-volume ratio, thus the majority of the active sensing components are embedded in the bulk, which inevitably limits the efficiency and sensitivity^23^. Furthermore, Pcs have been widely studied for functionalization of carbon nanotubes (CNTs) because they play important roles in enhancement of the performance of CNTs-based devices; they demonstrate attractive electronic and photoelectronic properties^21^,^24^. Recent reports show that MPc-CNTs hybrids exhibit enhanced electrochemical responses when compared to the use of CNTs or MPc alone^25^.

There are some reported studies on Pc modified electrodes for detection of DA^26^,^27^,^28^,^29^,^30^. For example, Martin *et al*.^26^ reported electrodeposited film of iron phthalocyanine (FePc) on glassy carbon and tin oxide electrodes for DA probing. Shaidarova *et al*.^27^ has reported determination of dopamine using electrode modified by a polyaniline film with an inclusion of copper (II) tetrasulfophthalocyanine. The nanocomposite modified electrode made possible the detection limit of DA to be 1 × 10^−8^ M. Naik *et al*.^29^ worked on surfactant induced iron (II) phthalocyanine modified carbon paste electrode for simultaneous detection of ascorbic acid, dopamine and uric acid. The modified electrode resolved the overlapped voltammetric responses of ascorbic acid, dopamine and uric acid in to three well-defined cyclic voltammetric peaks. These studies have employed metal nanoparticles (Cu, Ni, Co, or Fe) to doped Pc. However in this work, metal oxide nanoparticles (ZnO and Fe_3_O_4_) are used because: (a) there is paucity of literature on the use of MO doped Pc for DA detection, (b) synthesis route for MO nanoparticles are shorter and cheaper compared with their metal nanoparticles counterparts, (c) devices based on metal oxides nanostructures are cost-effective, highly sensitive due to the large surface-to-volume ratio of the nanostructure and additionally show excellent selectivity^8^ and this is of paramount importance to the present study because of interferences from other molecules (d) the need to explore the electron transfer mechanism of MO modified electrodes which is also fundamental to their application in sensors^12^, (e) study in our group have shown that MWCNT-Fe_2_O_3_ modified electrode gave excellent separation of DA signal from AA even at AA concentration 1000 times that of DA^12^, (f) the detection of many analytes at trace concentration using MO modified electrodes have been reported^12^,^31^.

In this work, electrocatalytic behaviour of ZnO and Fe_3_O_4_ nanoparticles doped phthalocyanines (Pc) and MWCNT on glassy carbon electrode (GCE) towards dopamine (DA) oxidation was investigated. Electrocatalytic oxidation of the analytes was successful on the modified electrodes, GCE-MWCNT/ZnO/29H, 31H-Pc was the best electrodes towards DA detection with very low detection limit that compared with literature, good sensitivity, resistance to electrode fouling, and excellent ability to detect DA without interference from AA signal. The sensor is easy to fabricate, cost effective and could be used for routine determination of dopamine in biological matrices. Electrocatalytic activities of the modified electrodes were linked with some molecular quantum chemical descriptors especially the frontier molecular orbitals and associated parameters.

## Experimental

### Materials and Reagents

Glassy carbon electrode (3 mm diameter) was purchased from CH Instrument USA. Polishing pads were obtained from Buehler, IL, USA and Alumina micro powder (1.0, 0.3 and 0.05 μm alumina slurries) was used for polishing the glassy carbon electrode (GCE). Pristine multi-walled carbon nanotubes (95% purity, 10–20 nm); Iron(III) chloride (FeCl_3_), zinc nitrate hexahydrate (Zn(NO_3_)_2_ · 6H_2_O), 29H,31H-Phthalocyanine (29H,31H-Pc), 2,3-Naphthalocyanine (2,3-Nc), dopamine hydrochloride, ascorbic acid and other reagents are of analytical grade and obtained from Sigma-Aldrich, Merck chemicals and LABCHEM respectively. Ultra-pure water of resistivity 18.2 MΩ was obtained from a Milli-Q Water System (Millipore Corp., Bedford, MA, USA) and was used throughout for the preparation of solutions. A phosphate buffer solution (PBS) of 7.0 was prepared with appropriate amounts of NaH_2_PO_4_ · 2H_2_O, Na_2_HPO_4_ · 2H_2_O, and H_3_PO_4_, and adjusted with 0.1 M H_3_PO_4_ or NaOH. Prepared solutions were purged with pure nitrogen to eliminate oxygen and prevent any form of external oxidation during every electrochemical experiment.

### Synthesis of Zinc Oxide (ZnO) Nanoparticles

Sodium hydroxide (NaOH) was dissolved in deionised water to a concentration of 1.0 M and the resulting solution was heated, under constant stirring, to the temperature of 70 °C. After achieving this temperature, a solution of 0.5 M Zn(NO_3_)_2_ · 6H_2_O was added slowly (drop wise for 60 minutes) into the NaOH aqueous solution under continuous stirring. In this procedure the reaction temperature was constantly maintained at 70 °C. The suspension formed with the dropping of 0.5 M Zn(NO_3_)_2_6H_2_O solution to the alkaline aqueous solution was kept stirred for two hours at 70 °C. The material formed was filtered and washed several times with deionised water. The washed sample was dried at 65 °C in the oven for 24 hours to obtain the dry powder^32^.

### Synthesis of Iron Oxide (Fe_3_O_4_) Nanoparticles

A mixture of FeCl_3_ stock solutions (30 mL) 2.0 M, Na_2_SO_3_ stock solution (20 mL) 1.0 M and concentrated ammonia (50.8 mL), diluted to a total volume of 800 mL was used. Fe^3+^ and SO_3_^2−^ were mixed; the colour of the solution changed from light yellow to red, indicating formation of a complex ion. The solution was quickly poured into the diluted ammonia solution under vigorous stirring, and the colour changed from red to yellow again. A black precipitate was formed and stirring continued for 30 minutes. After the reaction, the beaker containing the suspension was placed on a permanent magnet. The supernatant liquid was decanted and fresh deionised water was added into the beaker. This procedure was repeated several times until most of the ions in the suspensions were removed. The dry powder was obtained by filtration and drying at room temperature^33^.

### Preparation of MWCNT/Metal Oxide/Phthalocyanine Hybrids

Fe_3_O_4_ nanoparticles (2.5 mg) were dissolved in 1 mL DMF along with 2.0 mg of either of phthalocyanine 2,3-Nc or 29H,31H-Pc. The solution was allowed to mix with the aid of ultrasonication for 1 h. 2.0 mg of MWCNTs was dispersed in the DMF solution containing 2.5 mg of Fe_3_O_4_ nanoparticles and 2.0 mg of phthalocyanine under ultrasonication. The Fe_3_O_4_/phthalocyanine hybrids were allowed to adsorb onto MWCNTs by spontaneous adsorption and sonication for 5 h to give MWCNT/Fe_3_O_4_/phthalocyanine hybrids. MWCNT/ZnO/phthalocyanine hybrids were prepared using the same procedure.

### Equipment and Procedure

The Transmission electron microscopy (TEM) analysis was performed using Tecnai G2 Spirit FEI (USA). UV-vis analyses were carried out using UV-visible spectrophotometer (Agilent Technology, Cary series UV-vis spectrometer, USA). Electrochemical experiments were carried out using an Autolab Potentiostat PGSTAT 302 (Eco Chemie, Utrecht, and The Netherlands) driven by the GPES software version 4.9. Electrochemical impedance spectroscopy (EIS) measurements were performed with Autolab Frequency Response Analyser (FRA) software between 10 kHz and 1 Hz using a 5 mV rms sinusoidal modulation with the solution of the analyte at their respective peak potential of oxidation (vs. Ag|AgCl in sat’d KCl). A Ag|AgCl in saturated KCl and platinum wire were used as reference and counter electrodes respectively. A bench top Crison pH meter, Basic 20+ model, was used for pH measurements. All experiments were performed at 25 ± 1 °C while the solutions were de-aerated before every electrochemical experiment.

### Electrode Modification Procedure

Electrode modification was carried out using the drop-dry method. The glassy carbon electrode was cleaned by gentle polishing in aqueous slurry of alumina nanopowder on a silicon carbide-emery paper followed by a mirror finish on a Buehler felt pad. The electrode was further sonicated in double distilled water, and then absolute ethanol for 2 min to remove residual alumina particles that were trapped on the surface and dried at room temperature. Separate suspensions of metal oxide (MO) nanoparticles, phthalocyanines (Pc), MWCNT and MWCNT/MO/Pc hybrids were prepared in 1 mL of DMF and sonicated as described above. 10 μL drops of the prepared suspensions were dropped on the bare GCE and dried in an oven at 50 °C for 5 min to obtain GCE-MWCNT, GCE-ZnO and GCE-Fe_3_O_4,_ GCE-MWCNT/Fe_3_O_4_/2,3-Nc, GCE-MWCNT/Fe_3_O_4_/29H,31H-Pc, GCE-MWCNT/ZnO/2,3-Nc and GCE-MWCNT/ZnO/29H,31H-Pc electrodes.

### Preparation of the Dopamine Hydrochloride Injection Solution

2 mL of the drug (dopamine hydrochloride injection- Dopamine HCl-Fresenius^®^) sample was diluted to 100 mL with distilled de-ionized water. 2 mL of the diluted solution was pipette into six 50 mL volumetric flask and all except one were spiked with different concentration of standard dopamine solution, and made to volume with phosphate buffer pH 7.0. The concentration of each test aliquot solution was determined using square wave voltammetry. Two different injections from the same batch were analysed using the same procedure. The experiment was repeated 3 times for each sample.

## Results and Discussion

### UV-Vis, EDX, XRD and TEM Characterization

#### UV-Vis Results

**Figure 1:**
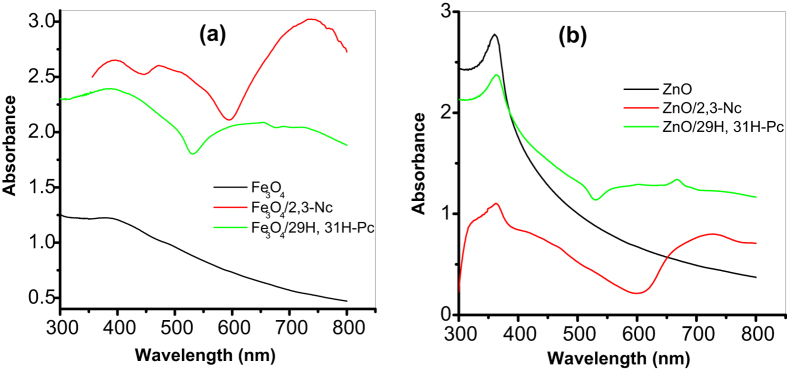
UV-Vis spectra of (**a**) Fe_3_O_4_, Fe_3_O_4_/2,3-Nc, Fe_3_O_4_/29H,31H-Pc and (**b**) ZnO, ZnO/2,3-Nc, ZnO/29H,31H-Pc in DMF solution.

Figure 1 shows the comparative UV-Vis absorption spectra for the metal oxide (MO) nanoparticles/phthalocyanines hybrids in DMF solution, confirming the formation of Fe_3_O_4_/2,3-Nc, Fe_3_O_4_/29H,31H-Pc, ZnO/2,3-Nc and ZnO/29H,31H-Pc composites. Phthalocyanines are organic semiconductors with important electrical properties applicable to sensor system. The UV-Vis spectrum of phthalocyanines originates from molecular orbitals within the aromatic 18π electron system^34^ and they have been considered as electrophotographic materials due to their absorption ability in the ultraviolet and visible region^35^. The evidence for the successful doping of 29H,31H-Pc and 2,3-Nc with Fe_3_O_4_ and ZnO is demonstrated by the change in the absorption bands as shown in the Figure. For example, Fe_3_O_4_ and ZnO nanoparticles showed absorption peaks at 397 nm and 370 nm (B band) respectively in the region of the visible spectrum which are in the range of 300 to 400 nm for transition metal oxide as reported in literature^36^,^37^. However after the phthalocyanine were doped with the nanoparticles there was a blue shift (386 nm) in absorption peak confirming formation of Fe_3_O_4_/29H,31H-Pc composite.

Appearance of a new peak at 665 nm and a red shift in absorbance peak at 376 nm (B-band) confirmed the formation of ZnO/29H,31H-Pc composite. Fe_3_O_4_/2,3-Nc and ZnO/2,3-Nc showed absorptions peaks at 369 nm and 362 nm respectively, which are lower compared with 397 nm and 370 nm for Fe_3_O_4_ and ZnO nanoparticles alone further confirming the successful formation of Fe_3_O_4_/2,3-Nc and ZnO/2,3-Nc composite. Broad absorption band for these two composites were also observed at 725 nm and 730 nm (Q-band) respectively. These characterized peaks of the Q-band have generally been interpreted in terms of the π-π* transitions of the π-electron on the phthalocyanine macrocycle^38^,^39^. Since phthalocyanines are found to be relatively good electron donors, Spadavecchia^40^ mentioned that the dispersion forces between adjacent molecules of phthalocyanine are produced by the delocalized π-electron system constructing a highly polarizable electronic cloud. As a result, low ionization energy favours the charge transfer interactions when electron acceptor molecules are adsorbed, hence the difference in the absorption spectra come from an increase of the density in the electronic levels available for π-π* transition. These electron cloud and charge transfer behaviour of Pc could also be responsible for the successful formation of the different MO/Pc composite synthesized in this study probably due to ionic and intermolecular interaction between the MO nanoparticles and the Pc molecules.

#### EDX and XRD Results

**Figure 2:**
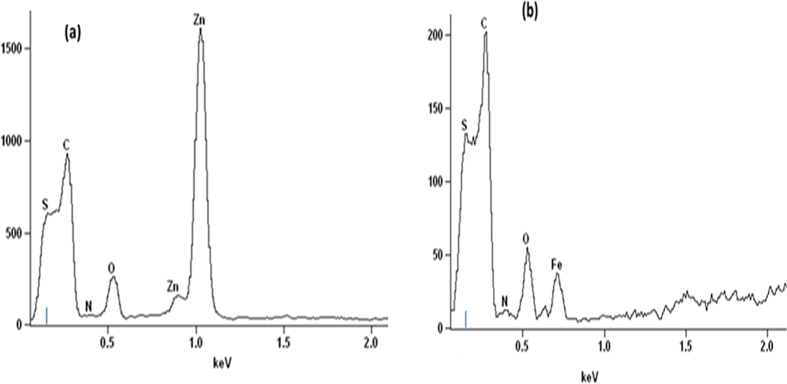
EDX spectra of (**a**) MWCNT/ZnO/Pc (**b**) MWCNT/Fe_3_O_4_/Pc.

The EDX spectra shown in Fig. 2 indicate the elemental composition of hybrids formed when phthalocyanines doped with metal oxides (Fe_3_O_4_, ZnO) were supported on MWCNTs. The presence of MWCNTs is confirmed by a carbon and sulfur, since the MWCNTs were purified and functionalized by acid mixture. The appearance of sulfur is due to sulfuric acid used during functionalization of MWCNTs. The presence of phthalocyanines is confirmed by a small peak of nitrogen while the zinc, oxygen and iron peaks further confirms the successful doping of Fe_3_O_4_ and ZnO. These results illustrates that MWCNTs was successfully modified with metal oxides doped phthalocyanine.

**Figure 3:**
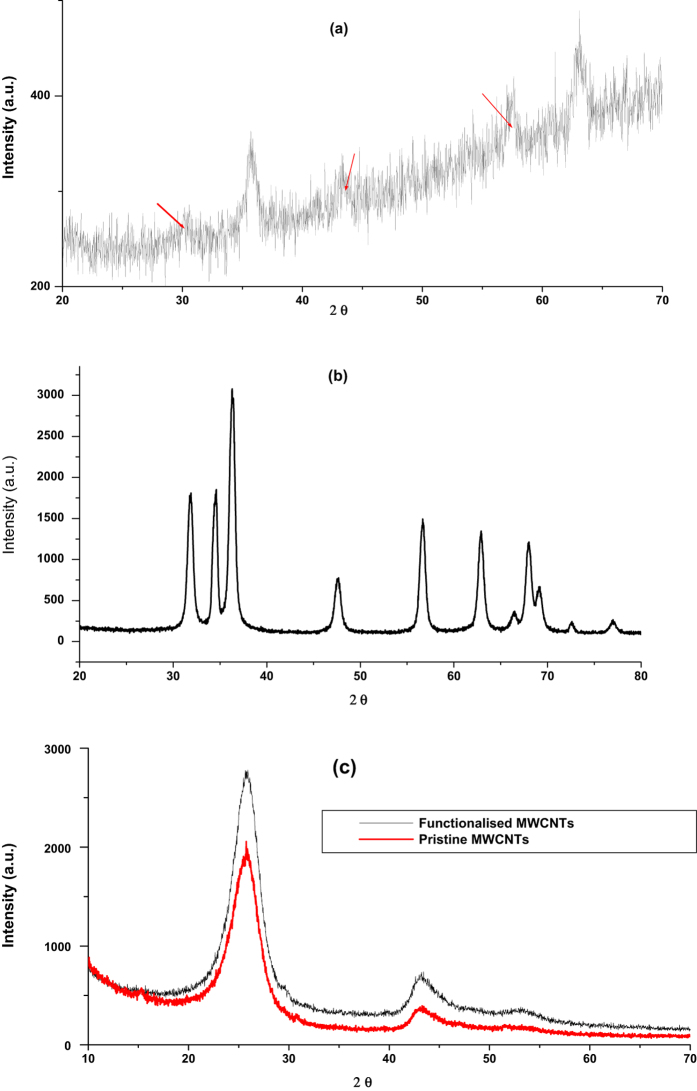
X-ray diffraction patterns of (**a**) Fe_3_O_4_ (**b**) ZnO (**c**) functionalized and pristine MWCNTs.

Figure 3(a) shows the XRD pattern of Fe_3_O_4_ nanoparticles. Five characteristic peaks for Fe_3_O_4_ occurred at 2θ of 30.01°, 35.6°, 43.27°, 57.02° and 62.76°. These peaks are in accordance with those reported in literature for pure Fe_3_O_4_ nanoparticles^37^,^41^. The corresponding indices of these diffraction peaks according to literature were (220), (311), (400), (511) and (440) for 30.01°, 35.6°, 43.27°, 57.02° and 62.76° respectively^41^,^42^,^43^. The average crystallite size obtained was 23.8 nm which was comparable to that obtained from TEM analysis. The diffraction peak at 2θ of 35.6° corresponds to the spinel phase of the Fe_3_O_4_ nanoparticles and the broadening of the diffracting bands indicates small particle size and ultrafine nanoparticles^37^,^43^. These data was in agreement with the XRD patterns of Fe_3_O_4_ nanoparticles reported in literature, therefore it confirms that the synthesized nanoparticles were pure Fe_3_O_4_ nanoparticles with spinel structure^41^,^44^.

Figure 3(b) illustrates the XRD pattern of ZnO nanoparticles. The diffraction peaks were obtained at 2θ of 31.77°, 34.52°, 36.29°, 47.49°, 56.65°, 62.88°, 66.14°, 67.93° and 69.11°, with corresponding indices of (100), (002), (101), (102), (110), (103), (200), (112) and (201) respectively^45^,^46^. The three sharp and intense diffraction peaks at 2θ of 31.77°, 34.52° and 36.29° indicates high crystallinity and the nanoparticles have polycrystalline structure^47^. Furthermore there were no observed peaks of any impurities indicating that nanoparticles synthesized were pure ZnO^48^.

According to literature the peaks for (100), (002) and (101) indices illustrates hexagonal ZnO of wurtzite structure^46^,^48^,^49^. The average crystallite size obtained was 14.2 nm which was comparable to that obtained from TEM analysis. Figure 3(c) shows the XRD pattern of functionalised and pristine MWCNTs. The three diffraction peaks observed at pristine MWCNTs also appeared at functionalized MWCNTs with enhanced intensity, indicating that MWCNTs were successfully functionalized with carboxyl groups, and also the MWCNTs structure was not destroyed. The diffraction peaks occurred at 2θ of 26.12°, 43.62° and 52.49° which corresponds to indices of (002), (100) and (101) respectively as reported in literature^13^,^50^. The broad peak (002) in the XRD pattern indicates the graphitic structure of CNTs^51^ which is the ordered arrangement of the concentric cylinders of graphitic carbon^52^. Therefore this confirmed the hexagonal structure of MWCNTs^50^.

#### TEM Characterization

**Figure 4:**
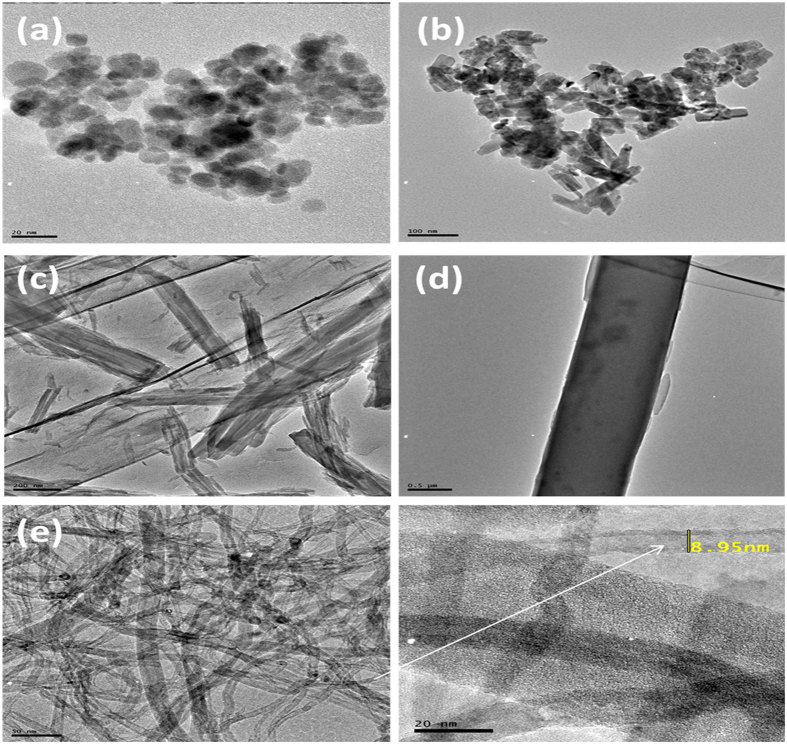
TEM images of (**a**) Fe_3_O_4_ NPs, (**b**) ZnO NPs, (**c**) 2,3-Nc, (**d**) 29H,31H-Pc, (**e**) MWCNT.

**Figure 5:**
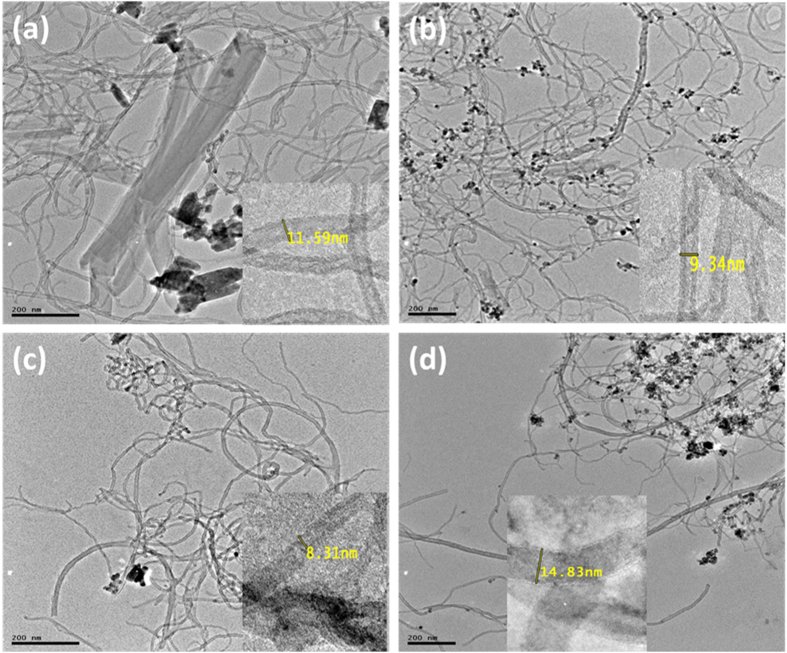
TEM images of (**a**) MWCNT/ZnO/2,3-Nc, (**b**) MWCNT/Fe_3_O_4_/2,3-Nc (**c**) MWCNT/ZnO/29H,31H-Pc and (**d**) MWCNT/Fe_3_O_4_/29H,31H-Pc.

The morphology and microstructures of prepared compounds and their composites were characterized using TEM. Figure 4 shows the TEM images of (a) Fe_3_O_4_ NPs, (b) ZnO NPs, (c) 2,3-Nc, (d) 29H,31H-Pc, and (e) MWCNT; while Fig. 5 shows the TEM images of (a) MWCNT/ZnO/2,3-Nc, (b) MWCNT/Fe_3_O_4_/2,3-Nc, (c) MWCNT/ZnO/29H,31H-Pc and (d) MWCNT/Fe_3_O_4_/29H,31H-Pc. The TEM images showed that Fe_3_O_4_ NPs were similar in shape, approximately spherical and they appeared to be almost uniformly distributed and less aggregated with the average diameter of 12 nm. The particle size was found to range from 6–23.7 nm. ZnO NPs were shaped like small rods and were aggregated with an average diameter of 26.1 nm. The aggregation might be due to the large surface area and high surface energy of ZnO NPs, which probably took place during the drying process^53^. The length size of the nanorods ranged from 20–78.6 nm. 2,3-Nc (c) and 29H,31H-Pc (d) images showed an irregularly shaped, small rod-like structures, or clustered flakes, none-uniform in both size and shape with a rough surface of 2,3-Nc (c) and a smooth surface of 29H,31H-Pc (d). The TEM image of MWCNTs (e) showed a tangled-net like structure of long tubes with a rather smooth surface. The presence of dark round spots show the cap ends of the tubes that were cut opened by acid treatment; they result from the oxidation of C-O, O–H and C–O groups to form COOH groups on the end caps of the CNTs. The MWCNTs showed a diameter of 8.95 nm.

The TEM images of MWCNT/ZnO/2,3-Nc (Fig. 5a) and MWCNT/ZnO/29H,31H-Pc (Fig. 5c) showed that ZnO nanoparticles and phthalocyanines combined into larger aggregates that were scattered and poorly dispersed on the MWCNTs for MWCNT/ZnO/2,3-Nc surface (Fig. 5a), and smaller particles were found distributed along the tubes for MWCNT/ZnO/29H,31H-Pc (Fig. 5c) but with tube diameters of 11.59 nm and 8.31 nm in both cases respectively as shown in inserted images. On the other hand the TEM images of (b) MWCNT/Fe_3_O_4_/2,3-Nc and (d) MWCNT/Fe_3_O_4_/29H,31H-Pc showed that Fe_3_O_4_ nanoparticles and phthalocyanine appeared to be almost uniformly dispersed that most of the nanoparticles were attached on the sidewalls of the MWCNTs. It can also be observed that the aggregation of Fe_3_O_4_ nanoparticles was minimal. The diameter of the tubes was 9.34 nm and 14.83 nm for (b) MWCNT/Fe_3_O_4_/2,3-Nc and (d) MWCNT/Fe_3_O_4_/29H,31H-Pc respectively.

#### Electrochemical Characterization of Modified Electrodes

**Figure 6:**
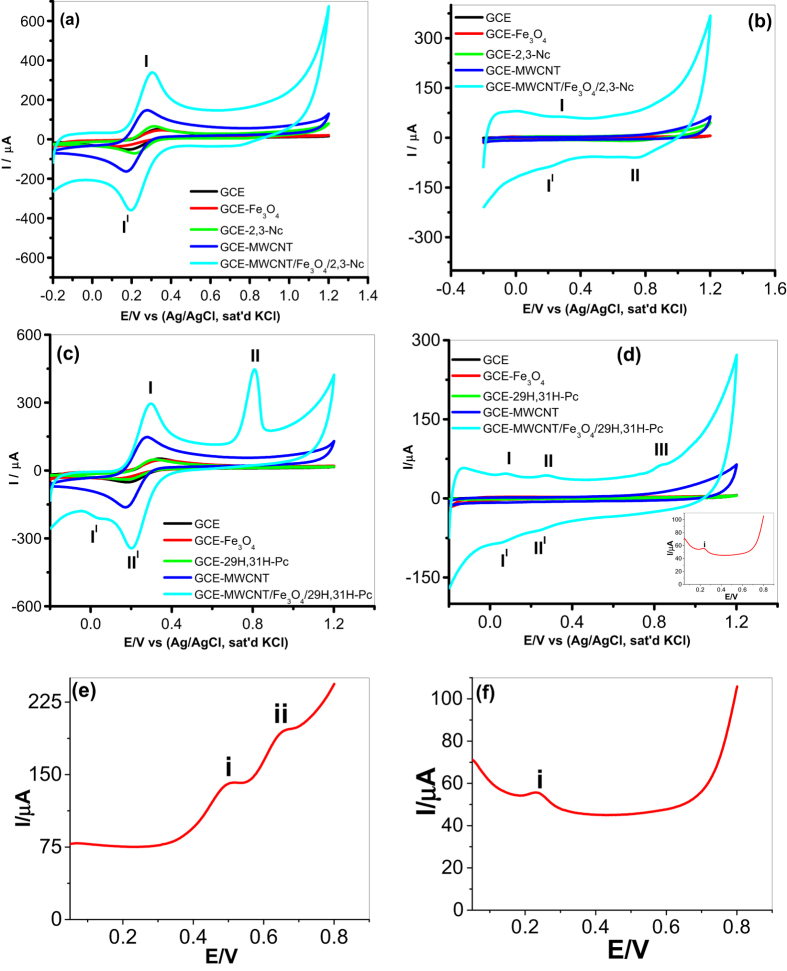
Comparative cyclic voltammetry evolutions of GCE and Fe_3_O_4_ NPs modified electrodes in 5 mM [Fe(CN)_6_]^4−^/[Fe(CN)_6_]^3−^ (**a**,**c**), and in pH 7.2 PBS (**b**,**d**). Linear sweep voltammograms of (**e**) GCE-MWCNT/Fe_3_O_4_/2,3-Nc and (**f**) GCE-MWCNT/Fe_3_O_4_/29H,31H-Pc.

**Figure 7:**
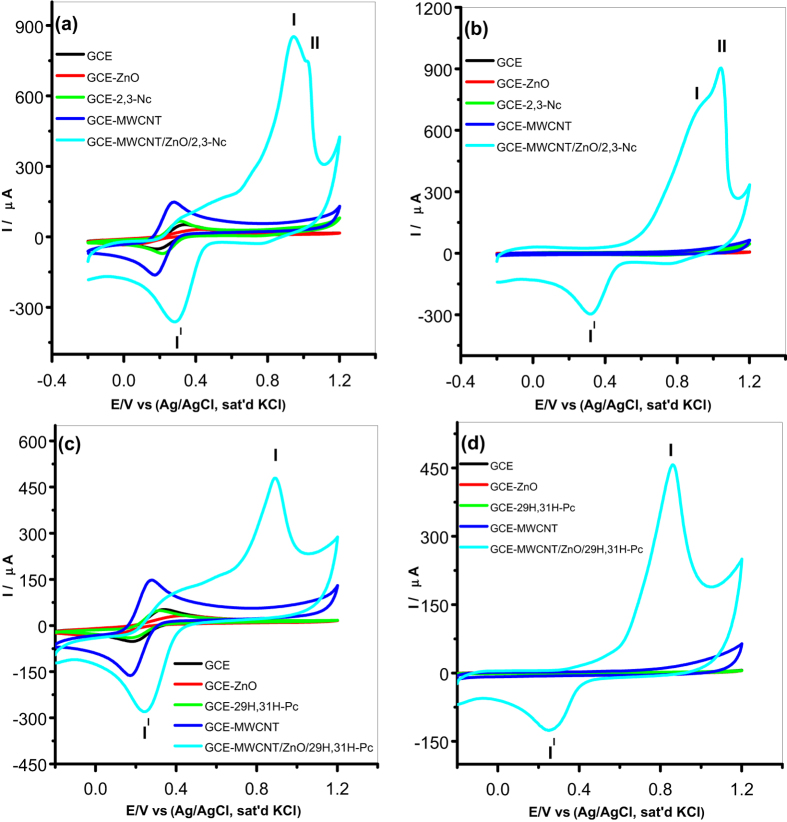
Cyclic voltammetry evolutions of GCE and ZnO NPs modified electrodes in 5 mM [Fe(CN)_6_]^4−^/[Fe(CN)_6_]^3−^ (**a**,**c**), and in pH 7.2 PBS (**b**,**d**).

Characterization of the modified electrodes was carried out in pH 7.2 PBS and 5 mM [Fe(CN)_6_]^4−^/[Fe(CN)_6_]^3−^ redox probe (scan rate 25 mV s^−1^) using cyclic voltammetry to determine their electron transport properties. Figures 6 and 7 illustrate comparative current response results obtained with the bare and modified electrodes. From Fig. 6, an enhanced current response for the GCE modified with 2,3-Nc, MWCNT, MWCNT/Fe_3_O_4_/2,3-Nc and MWCNT/Fe_3_O_4_/29H,31H-Pc in [Fe(CN)_6_]^4−^/[Fe(CN)_6_]^3−^ was observed as compared to the redox reaction on bare GCE and GCE modified with 29H,31H-Pc which showed a very small current response. Similar results were obtained on Fig. 7 when GCE was modified with 2,3-Nc, MWCNT, MWCNT/ZnO/2,3-Nc and MWCNT/ZnO/29H,31H-Pc, high current response was observed as compared to bare GCE and 29H,31H-Pc modified electrode. The GCE modified with ZnO and Fe_3_O_4_ NPs alone showed a drastic decrease in current response, this could be due to the formation of passive oxide layers of ZnO and Fe_3_O_4_ NPs, which were obstructive to the electron transfer activity^54^. Pairs of well-defined redox peaks were observed at **I** (0.2858 V), **I**^**I**^ (0.2174 V) and **I** (0.2834 V), **I**^**I**^ (0.2199 V) for Fig. 6a,c respectively. Similar pairs of redox peaks were observed in about the same redox potentials in Fig. 7(a,c). These redox peaks represent the [Fe(CN)_6_]^4−^/[Fe(CN)_6_]^3−^ redox process. Two additional peaks were discovered at **II** (0.8009 V) and **III** (0.05386 V) (Fig. 6c), these peaks might be associated with the oxidation of the Fe (II) to Fe (III) in Fe_3_O_4_ and reduction of Fe (III) to Fe (0). Similarly, two additional anodic peaks were detected on Fig. 7(a,c) at **II** (0.8864 V) and **II** (0.8766 V) respectively, which might be due to the electrochemical oxidation Zn (II) to Zn (III). These two peaks showed a high oxidation current that almost suppressed the anodic oxidation peak of [Fe(CN)_6_]^4−^/[Fe(CN)_6_]^3−^ redox process. Figure 6(e,f) shows linear sweep voltammograms of GCE-MWCNT/Fe_3_O_4_/2,3-Nc and GCE-MWCNT/Fe_3_O_4_/29H,31H-Pc respectively which substantiates the redox behavior of the electrodes.

Similarly, the GCE modified with MWCNT/Fe_3_O_4_/2,3-Nc, MWCNT/Fe_3_O_4_29H,31H-Pc (Fig. 6b,d) and MWCNT/ZnO/2,3-Nc, MWCNT/ZnO/29H,31H-Pc (Fig. 7b,d) in PBS electrolyte showed higher current response as compared with other modified electrodes. The oxidation peaks of Fe_3_O_4_ appeared for MWCNT/Fe_3_O_4_/2,3-Nc at **I** (0.2614 V) (Fig. 6b); and for MWCNT/Fe_3_O_4_/29H,31H-Pc at **I** (0.2600 V), **II** (0.30 V) and **III** (0.8009 V) (Fig. 6d). The peaks at around 0.26 and 0.3 correspond to the oxidation of Fe (II) to Fe (III), while the one around 0.8 V can be attributed to the oxidation of Fe (III) to FeOOH. Similar oxidation peaks were observed around 0.8–0.9 V (Fig. 7b,d) for ZnO oxidation. Zang^55^ mentioned that the electrochemical redox reaction of regular structured ZnO in batteries is not reversible and suggested that the reversibility of the redox reaction observed in PBS electrolyte might be attributed to the unique nanostructure of ZnO NPs fabricated. It is evident from the results in both electrolytes that there is combining cooperative effect between MO, Pc/Nc, and MWCNT that leads to enhance electron transport and high current response of the nanocomposite modified electrodes. This can mainly be due to the great surface area of MWCNT and Pc, and electrical conductivity of the MO and MWCNT NPs.

#### Electrochemical Impedance Spectroscopy Studies of Modified Electrodes

**Figure 8:**
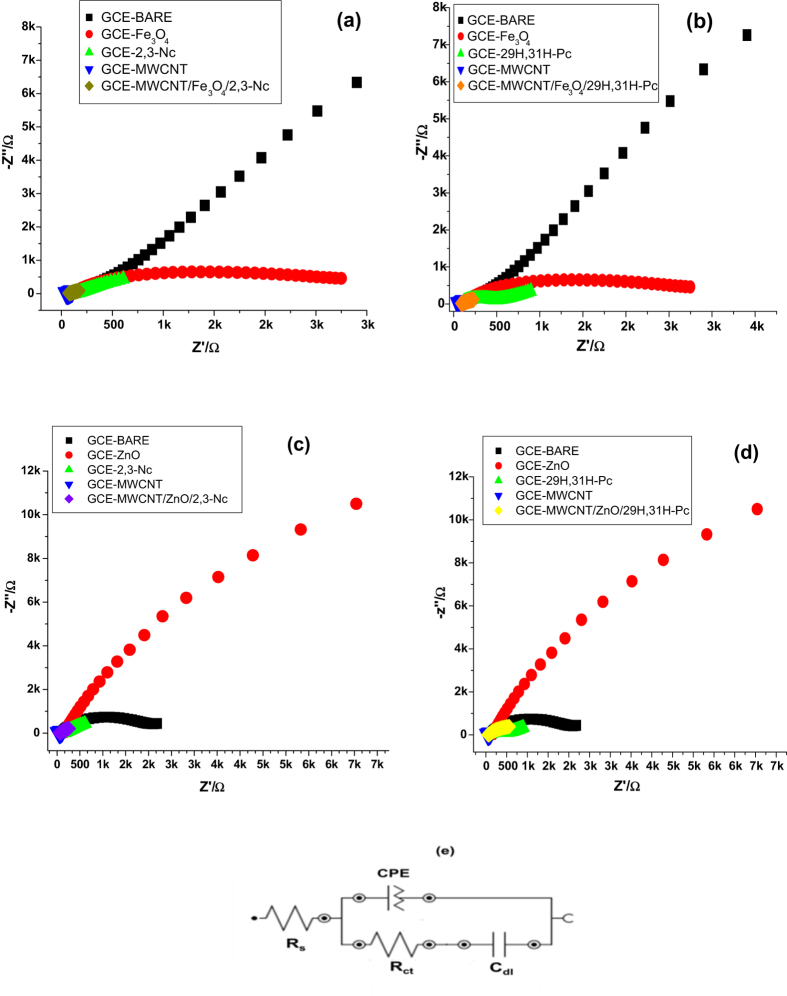
Typical Nyquist plots obtained for the bare and modified electrodes (**a**–**d**) in [Fe(CN)_6_]^4−^/[Fe(CN)_6_]^3−^ redox probe at fixed potential 0.22 V (vs Ag/AgCl, sat’d KCl). (**e**) Represents the circuit used in the fitting of the EIS data in (**a**–**d**).

The electrochemical impedance spectroscopy is a useful technique to investigate the electrode/solution interface properties. Figure 8 shows the Nyquist plots of the bare and modified electrodes in [Fe(CN)_6_]^4−^/[Fe(CN)_6_]^3−^ redox probe at a fixed potential of 0.22 V (vs Ag/AgCl, Sat’d KCl). The EIS data was recorded in the frequency range between 1 Hz to 10 kHz and the impedance parameters obtained are summarized in Table 1. Figure 8(e) represents the circuit used in the EIS fitting and it consists of solution resistance (R_s_), constant phase element (CPE) which relates to the porous nature of the electrode, charge transfer resistance (R_ct_) and double layer capacitance (C_dl_). The chi-square values, which are used to judge the goodness of EIS fittings and appropriateness of the adopted equivalent circuit and the percentage errors (in parentheses) associated with each circuit parameter are also listed in Table 1. The small chi-square values and percentage errors (Table 1) indicate that the experimental data are well fitted by the equivalent circuit. From the results obtained GCE-Fe_3_O_4_ and GCE-ZnO in Fig. 8 showed a semicircle domain. According to literature such behaviour demonstrates a charge transfer limited process^52^ and the diameter of the semicircle is equal to the charge transfer resistance^56^. Referring to impedance data in Table 1, the EIS of ZnO modified electrode showed a very large electron transfer resistance, this indicates that ZnO film was obstructive to the transfer of electrons of [Fe(CN)_6_]^4−^/[Fe(CN)_6_]^3−^ redox probe^57^, the same applied to Fe_3_O_4_. However MWCNT, MWCNT/Fe_3_O_4_/2,3-Nc, MWCNT/Fe_3_O_4_/29H,31H-Pc, MWCNT/ZnO/2,3-Nc and MWCNT/ZnO/29H,31H-Pc modified electrodes showed a very low charge transfer resistance, indicating that there was an electron conduction pathway formed between the composites and the electrolyte. These electrodes showed a linear plot which corresponds to the mass diffusion limited process^58^. Regarding the composites, the low charge transfer resistance might be due to the conductive nature of MWCNTs and atomic size (nano size) of the metals attached to the phthalocyanine and naphthalocyanine which accounts for the different rates of charge transfer and the other factor might be attributed to the axial ligands and substituents on the ring^59^. Therefore the oxidation of metal phthalocyanines may occur both at the metal and at the ring depending on the energies and proximity of the metal d and phthalocyanine ring orbitals^60^. These results are in good agreement with results obtained during CV studies.

### Electrocatalytic and Electroanalysis Studies

#### Electrocatalytic Oxidation of Dopamine (DA)

**Figure 9:**
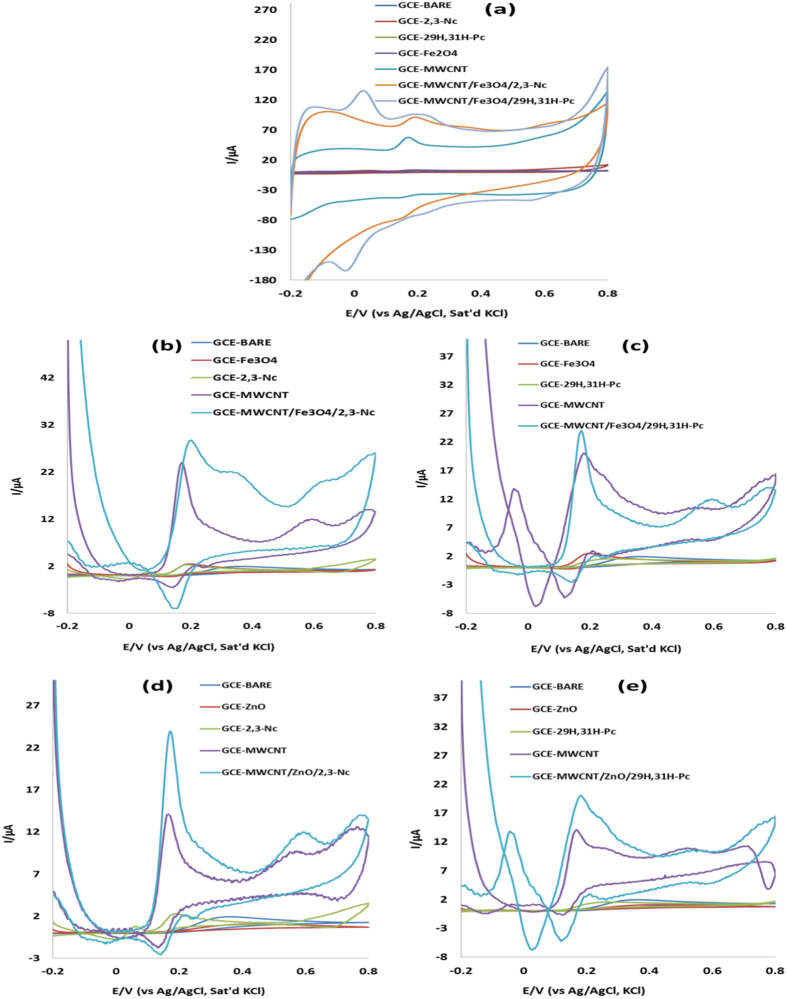
Cyclic voltammograms of bare GCE and GCE modified electrodes in pH 7.2 PBS containing 0.085 mM of DA (scan rate, 25mVs^−1^): (**a**) before background current subtraction, and (**b**–**e**) (after background current subtraction).

A series of CV experiments were conducted to investigate the electrocatalytic oxidation of DA in pH 7.2 PBS solution containing 0.085 mM of DA using the bare GCE and modified electrodes. Figure 9 presents the oxidation potentials and the comparative current response of the GCE electrode modified with Fe_3_O_4_ nanoparticles in the analyte. Except for Fig. 9(a), other figures present the voltammograms for the DA oxidation process after the background current subtraction. The voltammetric parameters such as I_pa_ and E_pa_ are summarized in Table 2. From the results obtained, broad ill-defined oxidation peaks of DA were observed for bare GCE, GCE-ZnO, and GCE-29H,31H-Pc. It is also interesting to note that the GCE-Fe_3_O_4_, GCE-ZnO and GCE-29H,31H-Pc gave low current response compared with the bare GCE. This may suggest that the Fe_3_O_4_ and ZnO nanoparticles film acted as passive layer obstructing the flow of current due to DA oxidation. However, an enhanced DA oxidation current response at lower oxidation potential was observed at the GCE-MWCNT/Fe_3_O_4_/2,3-Nc, and GCE-MWCNT/Fe_3_O_4_/29H,31H-Pc electrode (Fig. 9, Table 2). Similar trend was obtained for the ZnO nanoparticles GCE modified electrode where GCE-MWCNT/ZnO/2,3-Nc and GCE-MWCNT/ZnO/29H,31H-Pc electrodes gave the highest DA current at lower DA oxidation potential compared with the bare and GCE-MWCNT electrodes (Fig. 9, Table 2). The current was found to be 10 times higher for GCE-MWCNT/Fe_3_O_4_/29H,31H-Pc and GCE-MWCNT/ZnO/29H,31H-Pc than the bare electrode. Similarly, the current was approximately 15 and 12 times higher at GCE-MWCNT/Fe_3_O_4_/2,3-Nc and GCE-MWCNT/ZnO/2,3-Nc electrodes than the bare electrode. These developed GCE-MWCNT/MO/Pc sensors have demonstrated qualities of good electrochemical sensors because of (i) high current response and (ii) lower energy. The enhanced electrocatalysis of DA at the GCE-MWCNT/Fe_3_O_4_/2,3-Nc, GCE-MWCNT/Fe_3_O_4_/29H,31H-Pc, GCE-MWCNT/ZnO/2,3-Nc and GCE-MWCNT/ZnO/29H,31H-Pc electrodes indicates that the tri-composite of MO NPs, MWCNT and Pc promotes flow of electrons during DA oxidation as compared to MWCNT, MOs or Nc/Pc alone. Similar enhanced current response results have been obtained and reported in literature for modified electrodes^5^,^12^,^14^,^26^,^27^,^28^,^29^,^30^,^61^. The enhanced DA oxidation current at the MWCNT/MO/Pc modified electrodes can also be likened to other factors mentioned in literature such as the ease of diffusion of the DA through the porous-like MWCNT, MO^61^,^62^ and Pc film to the catalytic sites of the electrode, affinity between Fe^3+^ and DA^63^, the larger available surface area of the modified electrode due to the nanometric dimension of the nanotubes and, finally the strong combination tendency of the electron-rich oxygen atom of ZnO and Fe_3_O_4_ in MWCNT/MO/Pc film to DA by electrostatic interaction since DA is positively charged in neutral environment^64^ due to the protonation of the nitrogen atom on the molecule. Therefore further studies in this work were carried out using MWCNT/Fe_3_O_4_/2,3-Nc, MWCNT/Fe_3_O_4_/29H,31H-Pc, MWCNT/ZnO/2,3-Nc and MWCNT/ZnO/29H,31H-Pc electrodes.

#### Stability Studies

**Figure 10:**
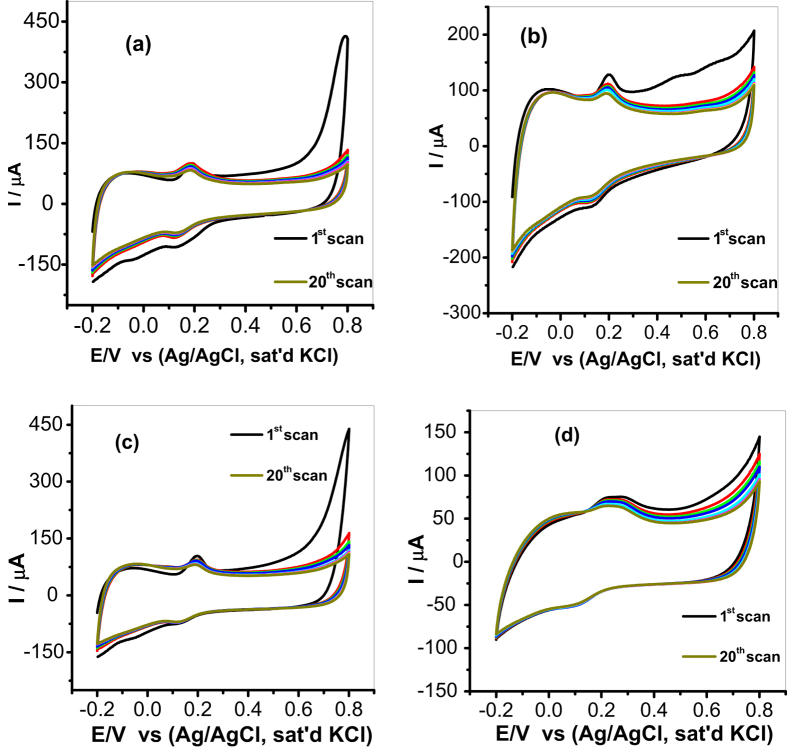
Current response (20 scans) of (**a**) MWCNT/Fe_3_O_4_/2,3-Nc, (**b**) MWCNT/Fe_3_O_4_/29H,31H-Pc, (**c**) MWCNT/ZnO/2,3-Nc and (**d**) MWCNT/ZnO/29H,31H-Pc electrodes in pH 7.2 PBS containing 0.085 mM of DA at scan rate of 25 mV s^−1^.

The stability of the fabricated sensors towards DA determination was investigated at 20 repeated CV scans in pH 7.2 PBS containing 0.085 mM of DA at scan rate of 25 mV s^−1^ as shown in Fig. 10. The stability was investigated using MWCNT/Fe_3_O_4_/2,3-Nc, MWCNT/Fe_3_O_4_/29H,31H-Pc, MWCNT/ZnO/2,3-Nc and MWCNT/ZnO/29H,31H-Pc modified electrodes. From the repeated scans, it was found that the peak current of the first cycle was more than the peak current of the second cycle and after the third cycle there was no significant drop in current response. This behavior was observed with all the four modified electrodes. Therefore the percentage current drop obtained was 5.65%, 5.88%, 5.56% and 5.26% for MWCNT/Fe_3_O_4_/2,3-Nc, MWCNT/Fe_3_O_4_/29H,31H-Pc, MWCNT/ZnO/2,3-Nc and MWCNT/ZnO/29H,31H-Pc modified electrodes respectively. Thus, the anodic and cathodic peak currents (I and I^I^) remained almost stable, indicating good electrodes stability during repeated cycles. These results suggest that there is some level of adsorption of DA at electrodes surface^65^. The adsorptive nature of electrodes might be due to the porous structured film of the CNTs on the electrodes^65^. The relative standard deviation (R.S.D.) of the fabricated sensors response to DA was 7.32%, 5.9%, 3.4% and 6.2% for MWCNT/Fe_3_O_4_/2,3-Nc, MWCNT/Fe_3_O_4_/29H,31H-Pc, MWCNT/ZnO/2,3-Nc and MWCNT/ZnO/29H,31H-Pc modified electrodes respectively, indicating that these electrodes are relatively stable and are not subjected to obvious surface fouling towards DA determination. The second reduction peak (II) was observed in Fig. 10(d), this peak might be attributed to the reduction of ZnOOH to Zn(OH)_2_ and ZnO respectively. GCE-MWCNT/ZnO/2,3-Nc showed a broad oxidation peak of DA as compared to other electrodes, however it gave the lowest RSD value indicating that it was the better performing and most stable electrode. The electrode can be used for the analysis of DA after storage in a refrigerator for up to two weeks without a significant change in its response.

#### The effect of scan rate (ν)

**Figure 11:**
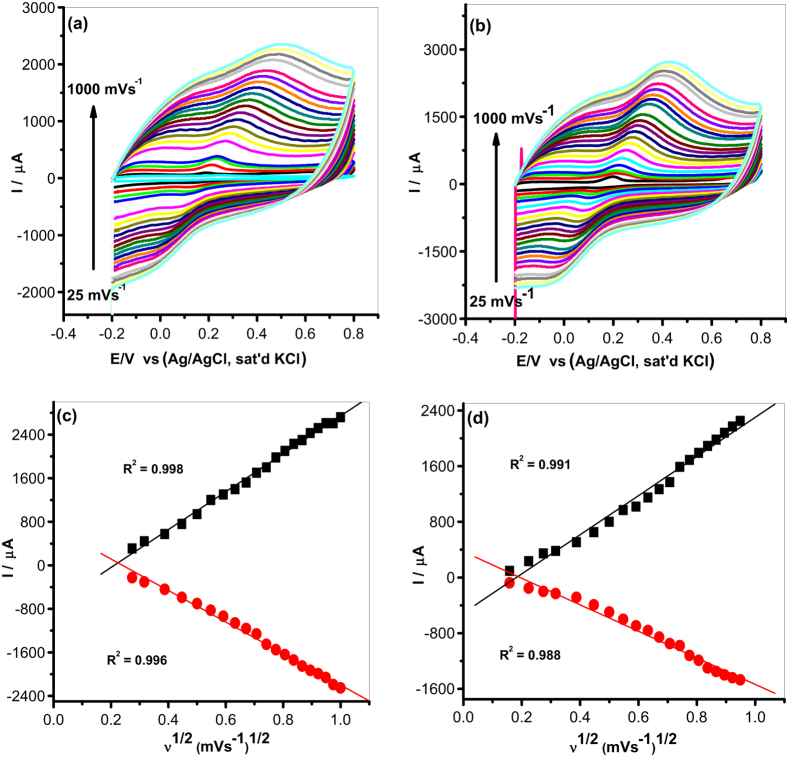
Cyclic voltammograms of modified electrodes: (**a**) MWCNT/Fe_3_O_4_/2,3-Nc (**b**) MWCNT/Fe_3_O_4_/29H,31H-Pc in pH 7.2 PBS containing 0.085 mM of DA at scan rate of 25–1000 mV s^−1^. (**c**,**d**) Are their respective plots of peak current vs. square root of scan rate.

**Figure 12:**
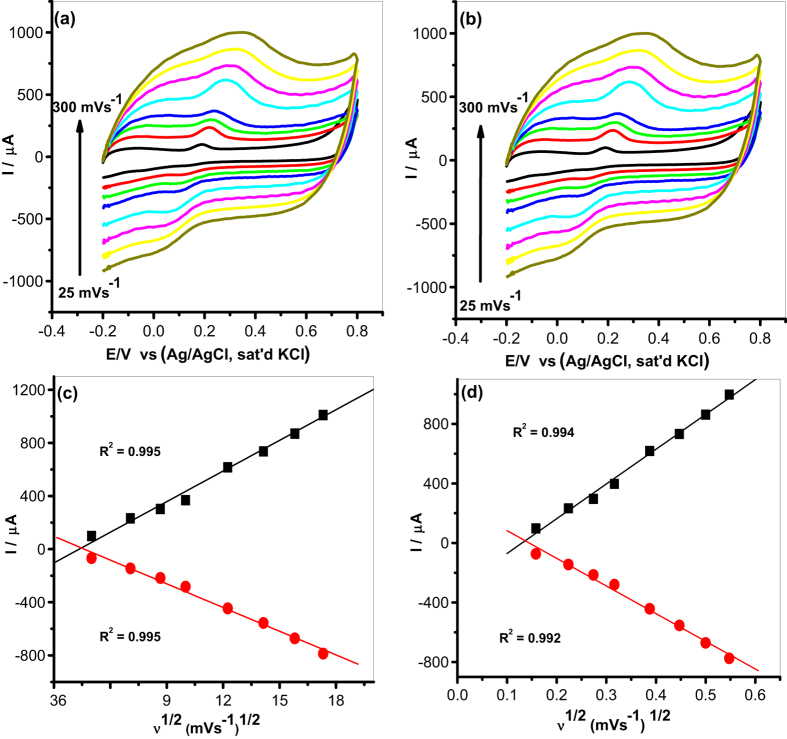
Cyclic voltammograms of modified electrodes: (**a**) MWCNT/ZnO/2,3-Nc (**b**) MWCNT/ZnO/29H,31H-Pc in pH 7.2 PBS containing 0.085 mM of DA at scan rate of 25–1000 mV s^−1^. (**c**,**d**) Are their respective plots of peak current vs. square root of scan rate.

Cyclic voltammetry was used to study the scan rate (ν) effect (range 25–1000 mV s^−1^) at a constant concentration of DA (0.085 mM) with electrodes modified with MWCNT/Fe_3_O_4_/2,3-Nc, MWCNT/Fe_3_O_4_/29H,31H-Pc, MWCNT/ZnO/2,3-Nc and MWCNT/ZnO/29H,31H-Pc as shown in Figs 11 and 12. The plots of peak current vs square root of scan rate showed a linear relationship. The oxidation peak currents were simultaneously increasing with increasing scan rate suggesting diffusion controlled process. Similarly, the anodic peak current (*I*_*pa*_) was directly proportional to the square root of scan rate (ν^1/2^) (Figs 11 and 12) but with negative intercept suggesting possibility of some adsorption intermediate in the process^65^. GCE-MWCNT/ZnO/2,3-Nc (Fig. 12a), GCE-MWCNT/ZnO/29H,31H-Pc (Fig. 12c) detect the presence of DA up to 300 mV s^−1^, from 350–1000 mV s^−1^ the oxidation peak current of DA became invisible. This might be due to electrode fouling effect. It could also suggest that the catalyst film on the electrode surface gets saturated and hence cannot detect the analyte over multiple cycles. Similar phenomenon have been observed by other workers and was attributed to factors such as method of electrode preparation, electrode conformation, the catalyst-analyte interaction, and porosity which play a significant role in the adsorption of the analyte on the catalyst film^65^.

The surface coverage (Γ) of DA on the surface of the modified electrode was calculated from the plot of peak current, *I*_*p*_ versus the square root of scan rate using Laviron equation^66^:


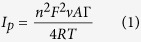


where *I*_*p*_ is the peak current in A, n is the number of electrons transferred, F is the Faraday constant (96 485. 3365 C mol^−1^), *ν* is the scan rate in Vs^−1^, A is the area of the electrode in cm^2^, Γ is the surface coverage of the analyte (mol cm^−2^), R is the ideal gas constant (8.3144621 J K^−1^ mol^−1^) and T is the temperature in K^66^. Assuming that n ≈ 2^62^, the calculated surface coverage of DA on electrodes modified with MWCNT/Fe_3_O_4_/2,3-Nc, MWCNT/Fe_3_O_4_/29H,31H-Pc, MWCNT/ZnO/2,3-Nc and MWCNT/ZnO/29H,31H-Pc were 1.462 × 10^−8^ mol cm^−2^, 1.34 × 10^−8^ mol cm^−2^, 1.054 × 10^−8^ mol cm^−2^ and 1.492 × 10^−8^ mol cm^−2^ respectively. Although it was deduced that the reaction process is diffusion controlled with some level of adsorption, it can be inferred that the analyte diffused significantly from the bulk solution to the electrode surface based on the values of DA surface coverage obtained.

The plots of peak potential (E_p_) versus the log of scan rate (log ν) (not shown) gave the Tafel values using the Tafel equation (Eq. 2)^67^,^68^:


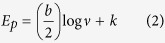


where *b* is the Tafel value, *k* is a constant. The Tafel values obtained for MWCNT/Fe_3_O_4_/2,3-Nc, MWCNT/Fe_3_O_4_/29H,31H-Pc, MWCNT/ZnO/2,3-Nc and MWCNT/ZnO/29H,31H-Pc modified electrodes were 811.6 mV dec^−1^, 631.2 mV dec^−1^, 137.8 mV dec^−1^, 259.6 mV dec^−1^ respectively. The high Tafel values could be due to electrode porosity, which lead to the adsorption of reactants or intermediates on the electrode surface^68^. In addition the data obtained from plots of peak potential (E_p_) versus the log of scan rate (log ν) showed a shift or an increase of peak potential as the log of scan rate increases. This variation of peak potential indicated that DA was oxidized by means of an adsorption process and the process was irreversible^69^.

#### Concentration study

**Figure 13:**
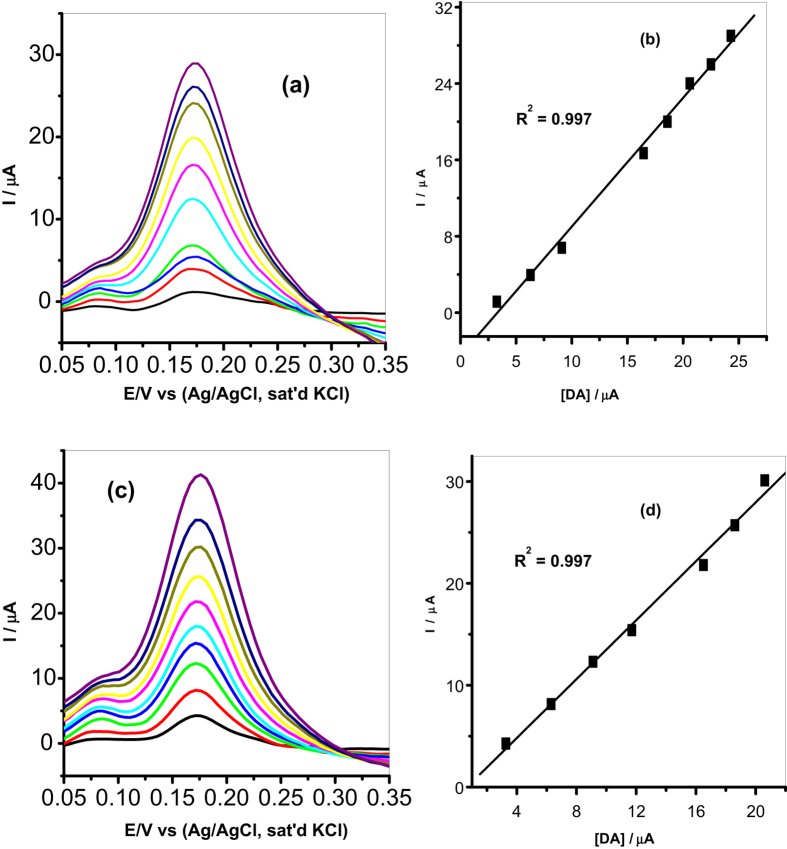
Differential pulse voltammograms of (**a**) MWCNT/Fe_3_O_4_/2,3-Nc and (**c**) MWCNT/Fe_3_O_4_/29H,31H-Pc modified electrodes in pH 7.2 PBS containing different concentrations of DA (3.27, 6.3, 9.11, 11.7, 14.2, 16.5, 18.6, 20.6, 22.5 and 24.3 μM). (**b**,**d**) Are their respective plots of peak current vs. DA concentration.

**Figure 14:**
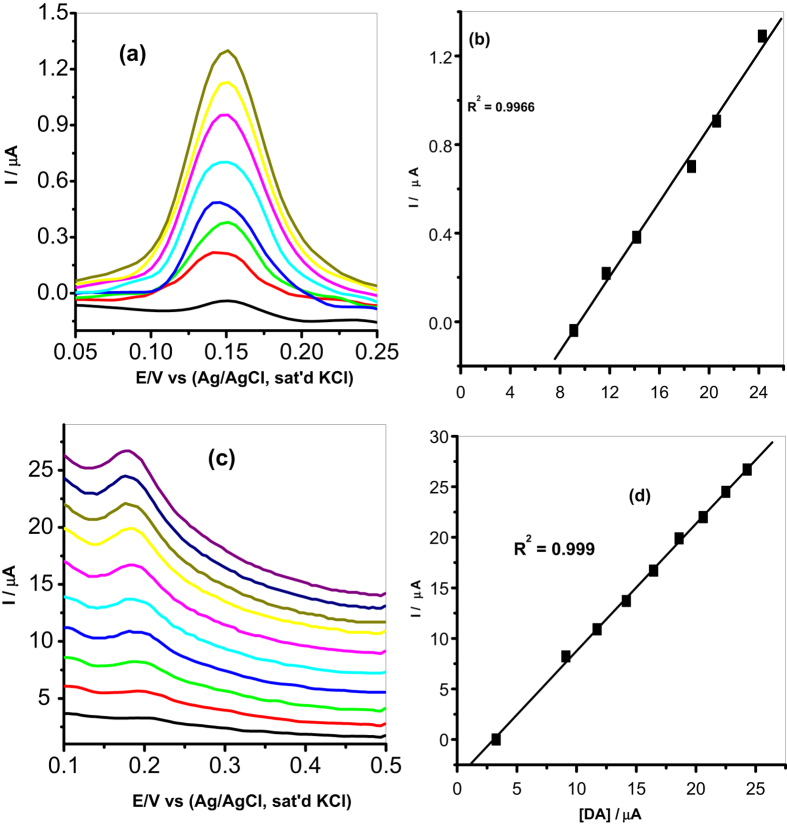
Differential pulse voltammograms of (**a**) MWCNT/ZnO/2,3-Nc and (**c**) MWCNT/ZnO/29H,31H-Pc modified electrodes in pH 7.2 PBS containing different concentrations of DA (3.27, 6.3, 9.11, 11.7, 14.2, 16.5, 18.6, 20.6, 22.5 and 24.3 μM). (**b**,**d**) Are their respective plots of peak current vs. DA concentration.

It is known that oxidation current depends on the concentration of the analyte, therefore because of the advantage of its good sensitivity, DPV was used to study the effect of concentration of DA oxidation on MWCNT/Fe_3_O_4_/2,3-Nc, MWCNT/Fe_3_O_4_/29H,31H-Pc, MWCNT/ZnO/2,3-Nc and MWCNT/ZnO/29H,31H-Pc modified electrodes. The experiment was carried out in pH 7.2 PBS containing different concentrations of DA ranging from 3.27–24.3 μM. The results showed that the oxidation currents of DA at the surface of the modified electrodes were increasing with DA concentration. The linear variation of peak current with concentration is shown in Figs 13 and 14.

The limit of detection (LOD) of the modified electrodes was calculated using 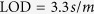
70 where s is the relative standard deviation of the intercept, m is the slope of the linear current versus the concentration of the analyte. The obtained detection limits were, 1.77, 1.35, 2.34 and 0.75 μM for MWCNT/Fe_3_O_4_/2,3-Nc, MWCNT/Fe_3_O_4_/29H,31H-Pc, MWCNT/ZnO/2,3-Nc and MWCNT/ZnO/29H,31H-Pc modified electrodes respectively.

The limit of detection obtained in this work compared favourably with others reported in literature^12^,^27^,^52^,^59^,^61^. From the results obtained it can be seen that MWCNT/ZnO/29H,31H-Pc modified electrode gave a low limit of detection with low sensitivity of 1.26 μA/μM as compared to the high sensitivity of MWCNT/Fe_3_O_4_/2,3-Nc (1.35 μA/μM) and MWCNT/Fe_3_O_4_/29H,31H-Pc (1.45 μA/μM) modified electrodes. However, it has been reported that the limit of detection does not only depend on the sensitivity of a sensor. Fialkov *et al*.^71^ mentioned that sensitivity has often been mistakenly referred to as the ability to achieve low limits of detection, meaning that the more sensitive an electrode is, the low limit of detection can be achieved. He defined sensitivity as the change in signal versus the change in concentration of the analyte, or it can be defined as the slope of the calibration curve in an analysis. The sensitivity of a sensor depends widely on the reactive surface area of a sensor and the catalytic material used to modify an electrode^72^. In fabrication of a sensor, a limit of detection is more important to investigate the performance of a sensor. A sensor with a high sensitivity won’t necessarily equate to a low limit of detection, meaning a highly sensitive sensor may have a high background noise level which might not be a problem at higher concentrations, but at lower concentrations excessive noise can hinder the good measurements of detection limits^72^. Therefore other than increasing sensitivity, a low detection limit can be achieved by reducing matrix interference (increasing selectivity) and high background noise^71^.

#### Interference Study

**Figure 15:**
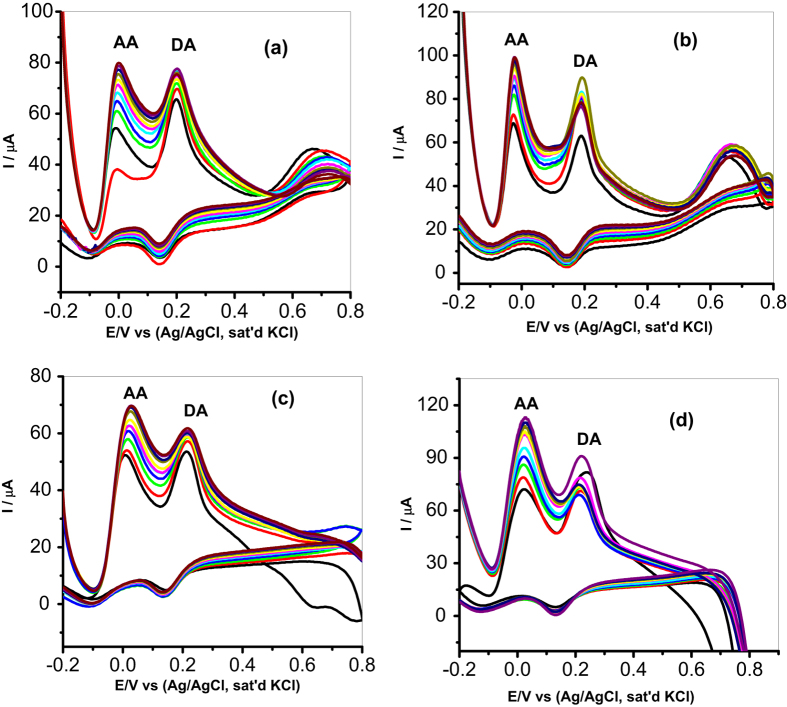
Cyclic voltammograms of binary mixture of DA and AA at (**a**) MWCNT/Fe_3_O_4_/2,3-Nc, (**b**) MWCNT/Fe_3_O_4_/29H,31H-Pc, (**c**) MWCNT/ZnO/2,3-Nc and (**d**) MWCNT/ZnO/29H,31H-Pc with constant concentration of DA (0.085 mM) and increasing concentration of AA (0.031, 0.058, 0.081, 0.1, 0.12, 0.13, 0.14, 0.16, 0.17, 0.18 mM).

DA coexists with other molecules in our biological fluids. AA is an electroactive species known to interfere in DA analysis in biological fluids. The basal concentration of DA is 0.01–1 μM, while the concentration of AA and UA is 100–1000 times higher than that of DA. Hence simultaneous detection of these analytes and the ability to detect DA selectively is important in sensor fabrication. AA is mostly oxidized at similar potentials with DA which results in overlapping of signals on most modified electrodes. The selectivity and sensitivity of MWCNT/Fe_3_O_4_/2,3-Nc, MWCNT/Fe_3_O_4_/29H,31H-Pc, MWCNT/ZnO/2,3-Nc and MWCNT/ZnO/29H,31H-Pc modified electrodes towards DA was investigated using CV with increasing concentration of AA (0.031, 0.058, 0.081, 0.1, 0.12, 0.13, 0.14, 0.16, 0.17, 0.18 mM) while the concentration of DA (0.085 mM) was kept constant. In Fig. 15, two well separated oxidation peaks of AA and DA were observed for all the four electrodes. It was observed that up to 30-fold excess of AA there was no significant interference between AA and DA peaks separations. Oxidation peak potentials and the difference between the two peak potentials are summarized in Table 3. The difference between oxidation peak potentials for all the four electrodes was high enough to distinguish DA from AA. The height and the amplitude of the oxidation peaks corresponding to AA increased proportionally with the concentration of AA. At MWCNT/Fe_3_O_4_/2,3-Nc, MWCNT/ZnO/2,3-Nc and MWCNT/ZnO/29H,31H-Pc modified electrodes there was a shift in peak potentials of DA as compared to original peak potentials in the catalysis studies. Although it was mentioned that in neutral pH, AA is negatively charged and DA is positively charged^61^,^62^,^64^, therefore the substantial shift in peak potentials could be related to the electrostatic repulsion between negatively charged AA, the electron reach oxygen atom of the MO nanoparticles and the negatively charged COO^−^ functionalized MWCNT. It can be seen that the anodic peak signals of AA and DA were independent from each other; therefore the modified electrodes were able to adequately identify the two analytes. The height and amplitude of the peak corresponding to DA also increase proportionally with the DA concentration. In fact, in all the concentrations of the DA studied there was no detectable interference of the AA, the signal of the AA was about 200 mV away from that of the DA. However MWCNT/Fe_3_O_4_/2,3-Nc and MWCNT/ZnO/2,3-Nc modified electrodes showed better results in terms of peak separation. This might be due to the extension of π-electron systems of Ncs which facilitates its improved repulsion of the interfering species as compared to Pcs analogues^73^,^74^.

#### Application of modified electrode in real sample analysis

**Figure 16:**
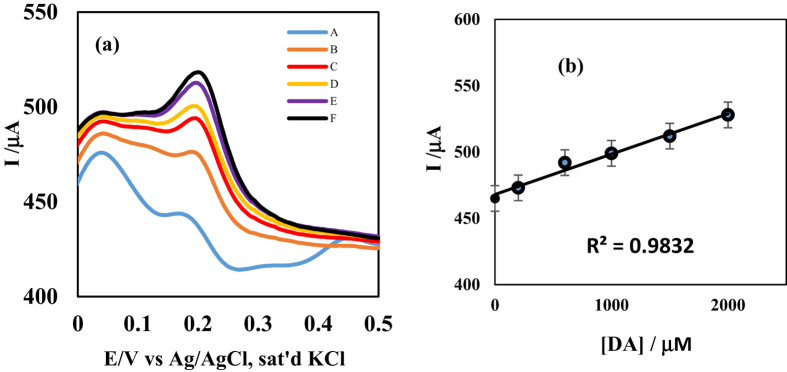
(**a**) Square wave voltammetry of modified MWCNT/ZnO/29H,31H-Pc-GCE in **A** (dil. DA drug alone), **B** (DA drug + 2 mL, 2 × 10^−4^ M DA), **C** (DA drug + 2 mL 6 × 10^−4^ M DA), **D** (DA drug + 2 mL 10 × 10^−4^ M DA), **E** (DA drug + 2 mL 15 × 10^−4^ M DA) and **F** (DA drug + 2 mL 20 × 10^−4^ M DA); (**b**) the standard addition method linear plot of current versus concentration of DA.

A square wave voltammetric assay of dopamine present in a dopamine hydrochloride injection, with dopamine content of 200 mg/5 mL (i.e. 40 mg mL^−1^) was carried out using MWCNT/ZnO/29H,31H-Pc (Fig. 16) and MWCNT/Fe_3_O_4_/29H,31H-Pc (not shown) modified GC electrodes. The concentration found in each dopamine drug was approximately within the range of the labelled amount, with an average recovery (n = 3) of 119.8 ± 0.06% and 101.3 ± 0.03% for MWCNT/ZnO/29H,31H-Pc and MWCNT/Fe_3_O_4_/29H,31H-Pc modified GC electrodes respectively suggesting the sensitivity of the developed sensors towards dopamine determination in real life samples. The result is presented in Table 4.

#### Relating electrocatalytic properties of modified electrodes with quantum chemical descriptors of 29H,31H-Pc and 2,3-Nc

Isaacs *et al*.^75^ related electrocatalytic and redox activities of Co-Pc and Co-Nc to experimentally observed electronic spectra of the complexes. In their work, the authors reported nearly the same values of lowest unoccupied molecular orbital energy (E_LUMO_) for the two complexes, higher value of highest occupied molecular orbital energy (E_HOMO_) and lower value of HOMO-LUMO energy gap (∆E_L-H_) for Co-Nc compared to Co-Pc. Similar results have also been reported earlier based on experimental and theoretical semiempirical calculations^76^. These and our previous knowledge of frontier molecular orbital (FMO) energies and electron density distributions of Pcs and Ncs motivated us to explore possible correlations between theoretical quantum chemical parameters of 29H,31H-Pc and 2,3-Nc and the performance of our fabricated electrodes. Even though the composite electrode systems of the form GCE-MWCNT/metal oxide/macrocycle used in the experimental sections could not be modeled in the present work, attempt was made to correlate the observed good electrocatalytic behavior of the electrodes with molecular structure and FMO indices of the utilized macrocycles (29H,31H-Pc and 2,3-Nc).

**Figure 17:**
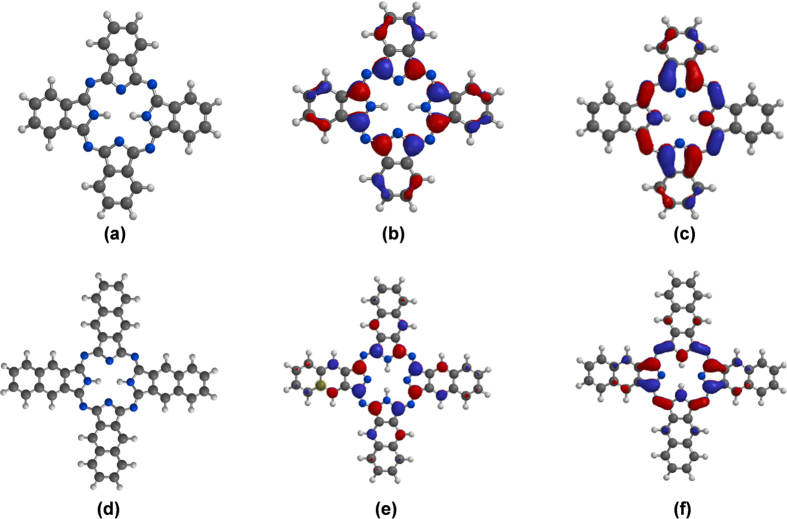
Optimized structures (**a**,**d**), HOMO (**b**,**e**) and LUMO (**c**,**f**) electron density isosurfaces of 29H,31H-Pc and 2,3-Nc respectively^77^.

Figure 17 shows the optimized molecular structures, HOMO and LUMO electron density isosurfaces of 29H,31H-Pc and 2,3-Nc. The apparent planar geometries, widely delocalized HOMO and LUMO electron densities, and presence of σ and π orbitals in the FMOs are favourable to electron-transport processes between these macrocycles, the metal oxides (Fe_3_O_4_ and ZnO) used in the composite electrode systems and the analyte (DA). Computational details and extensive explanations of other quantum chemical parameters on 29H,31H-Pc and 2,3-Nc including Mulliken atomic charges are contained in our previous work^77^.

Table 5 shows selected quantum chemical descriptors of 29H,31H-Pc and 2,3-Nc obtained using the B3LYP/6-31G(d,p) model chemistry and reported elsewhere^77^. The results revealed high values of E_HOMO_ and low values of E_LUMO_ for the compounds. Generally, a high value of E_HOMO_ connotes high tendency of a molecule to donate its HOMO electron to a suitable accepting orbital while a low value of E_LUMO_ implies good propensity of a molecule to accept electron into its LUMO from a suitable donor orbital, and vice versa. The results in Table 5 support better electron transport properties of 29H,31H-Pc and 2,3-Nc and their good electrocatalytic behaviours. It was also observed that 29H,31H-Pc is more disposed to electron acceptance than 2,3-Nc based its lower value of E_LUMO_ and higher value of electronegativity (χ).

If it is assumed that oxidation of DA to dopamine-o-quinone occurred according to the equation^78^:


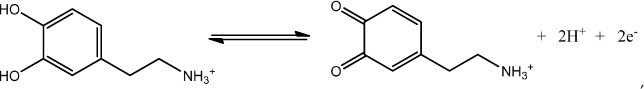


then, 29H,31H-Pc with better electron accepting ability (lower E_LUMO_ and higher χ) may show better electrocatalytic property than 2,3-Nc towards DA oxidation. In other words, an electrode system containing a better electron accepting specie as a catalyst may show better detection of DA. This assumption is in agreement with our observed (experimental) LOD values in which electrode systems containing 29H,31H-Pc exhibited lower LODs than those containing 2,3-Nc.

## Conclusions

The study described successful modification of GCE electrode with MWCNT, ZnO, Fe_2_O_3_, Pc, Nc, MWCNT/Fe_3_O_4_/2,3-Nc, MWCNT/Fe_3_O_4_/29H,31H-Pc, MWCNT/ZnO/2,3-Nc and MWCNT/ZnO/29H,31H-Pc nanomaterials. The electrochemical measurements indicated that MWCNT/Fe_3_O_4_/2,3-Nc, MWCNT/Fe_3_O_4_29H,31H-Pc MWCNT/ZnO/2,3-Nc and MWCNT/ZnO/29H,31H-Pc hybrids have pronounced electrocatalytic properties and enhanced dopamine current response on GCE compared to other catalyst studied. The enhanced current response is associated with the excellent strong super paramagnetic properties of Fe_3_O_4_ and high electrical conductivity of MWCNTs, ZnO and phthalocyanines. All the four electrodes showed a good stability with low current drop (<10%) towards DA oxidation. GCE-MWCNT/ZnO/29H,31H-Pc was the best electrodes towards DA detection with very low detection limit that compared with literature, good sensitivity, resistance to electrode fouling, and excellent ability to detect DA without interference from AA signal. Electrocatalytic oxidation of DA on GCE-MWCNT/ZnO/29H,31H-Pc electrode was diffusion controlled but characterized with some adsorption of electro-oxidation reaction intermediates products. The MWCNT/ZnO/29H,31H-Pc and MWCNT/Fe_3_O_4_/29H,31H-Pc GC modified electrodes has also proven to be a potential sensor for dopamine detection in real sample analysis. The relatively high-lying highest occupied molecular orbitals and low-lying lowest unoccupied molecular orbitals of 29H,31H-Pc and 2,3-Nc may be partly responsible for their great electrocatalytic activities. The better sensing properties of 29H,31H-Pc containing modified electrodes towards DA might be connected with the better electron-accepting ability of 29H,31H-Pc (compared to 2,3-Nc).

## Additional Information

**How to cite this article**: Mphuthi, N. G. *et al*. Phthalocyanine Doped Metal Oxide Nanoparticles on Multiwalled Carbon Nanotubes Platform for the detection of Dopamine. *Sci. Rep.* **7**, 43181; doi: 10.1038/srep43181 (2017).

**Publisher's note:** Springer Nature remains neutral with regard to jurisdictional claims in published maps and institutional affiliations.

## Figures and Tables

**Table 1:** Impedance data obtained for bare and modified electrodes in [Fe(CN)_6_]^4−^/[Fe(CN)_6_]^3−^ redox probe at fixed potential 0.22 V (vs Ag/AgCl, sat’d KCl).

Fabricated Electrodes	Impedance parameters				
	R_s_ (Ω cm^2^)	R_ct_ (Ω cm^2^)	C_dl_ (μF)	CPE	Chi-square
BARE	54.3 (8.332)	1211 (2.547)	4.46 (2.66)	0.533E-3 (7.029)	0.09217
Fe_3_O_4_	28.06 (32.709)	981 (5.612)	2.06 (7.026)	0.31E-3 (3.391)	0.369
ZnO	83.5 (2.521)	3890 (5.747)	3.61 (6.365)	0.276E-4 (3.342)	0.06135
2,3-Nc	57.3 (20.32)	85.5 (14.5)	70.0 (29.00)	0.63E-3 (0.702)	0.00497
29H,31H-Pc	29.95 (33.77)	178.6 (4.411)	1.177 (5.905)	0.869E-3 (1.876)	0.04612
MWCNT	24.56 (15.976)	33.1 (11.367)	0.325 (24.346)	0.409E-2 (3.346)	0.0427
MWCNT/Fe_3_O_4_/2,3-Nc	38.8 (0.00)	39.1 (3.851)	1000 (0.00)	0.258E-2 (24.7)	0.202
MWCNT/Fe_3_O_4_/29H,31H-Pc	78.8 (3.663)	53.4 (13.503)	126.0 (13.347)	0.263E-2 (5.558)	0.0851
MWCNT/ZnO/2,3-Nc	53.9 (5.285)	35.2 (12.997)	142.6 (9.474)	0.144E-2 (6.770)	0.1321
MWCNT/ZnO/29H,31H-Pc	41.1 (6.33)	78.7 (6.923)	30.988 (7.729)	0.7112E-3 (4.191)	0.117

The values in brackets represent the percentage errors of the data fitting.

**Table 2:** Cyclic voltammetric parameters obtained for bare and modified electrodes in pH 7.2 PBS containing 0.085 mM of DA at scan rate 25 mV s^−1^.

Fabricated GC electrodes	Cyclic voltammetric parameter	
	I_pa_ (μA)	E_pa_ (V)
BARE	1.94	0.256
Fe_3_O_4_	1.47	0.196
ZnO	0.21	0.173
2,3-Nc	2.3	0.192
29H,31H-Pc	1.53	0.266
MWCNT	14.4	0.176
MWCNT/Fe_3_O_4_/2,3-Nc	29	0.201
MWCNT/Fe_3_O_4_/29H,31H-Pc	20	0.186
MWCNT/ZnO/2,3-Nc	23.7	0.168
MWCNT/ZnO/29H,31H-Pc	19.8	0.174

**Table 3:** Cyclic voltammetric parameters of binary mixture of DA and AA obtained for modified electrodes with constant concentration of DA (0.085 mM) and increasing concentration of AA (0.031, 0.058, 0.081, 0.1, 0.12, 0.13, 0.14, 0.16, 0.17, 0.18 mM).

Fabricated GC electrodes	Anodic peak potentials (V)		Peak potential separation (V)
	AA	DA	
MWCNT/Fe_3_O_4_/2,3-Nc	−0.01	0.202	0.192
MWCNT/Fe_3_O_4_/29H,31H-Pc	−0.022	0.189	0.167
MWCNT/ZnO/2,3-Nc	0.021	0.218	0.197
MWCNT/ZnO/29H,31H-Pc	0.024	0.218	0.194

**Table 4:** Results of detection of DA in dopamine hydrochloride injections (n = 3) at MWCNT/ZnO/29H,31H-Pc and MWCNT/Fe_3_O_4_/29H,31H-Pc^a^.

Electrode	Added (mg/mL)	Detected (mg/mL)	Recovery (%)	RSD %
Sample 1	40	47.50 (34.50)^a^	119.8 (86.40)^a^	0.06 (0.69)^a^
Sample 2	40	40.15 (40.54)^a^	100.4 (101.30)^a^	0.09 (0.03)^a^

**Table 5:** Quantum chemical parameters for 29H,31H-Pc and 2,3-Nc^77^.

Macrocycle/Parameters	E_HOMO_ (eV)	E_LUMO_ (eV)	∆E_L-H_ (eV)	ϰ (eV)
29H,31H-Pc	−4.98	−2.83	2.15	3.91
2,3-Nc	−4.55	−2.76	1.79	3.66
